# Artificial intelligence ethics: automation of medical publishing

**DOI:** 10.3399/BJGPO.2026.0048

**Published:** 2026-04-22

**Authors:** Patrick Burch, Hajira Dambha-Miller

**Affiliations:** 1 Centre for Primary Care and Health Services Research, University of Manchester, Manchester, UK; 2 BJGP Open, Royal College of General Practitioners, London, UK; 3 Primary Care Research Centre, University of Southampton, Southampton, UK

**Keywords:** Ethics, Research methods, General practitioners, Primary Healthcare

Artificial intelligence is rapidly becoming embedded across the lifecycle of medical research and publishing, from literature-searching and data analysis to manuscript drafting, peer review, and editorial decision-making. These developments promise efficiency and scale, but they also raise ethical questions that extend beyond technical accuracy or the risk of error. AI systems reshape how knowledge is produced, interpreted, and trusted, introducing new challenges for authorship, accountability, transparency, and equity. A recent survey suggests that 69% of researchers have experimented with generative AI tools in some aspect of their workflow, and several major publishers have reported a sharp rise in manuscripts that disclose AI assistance since 2023.^1,2^ The scale and speed of adoption mean that governance is struggling to keep pace. As their use becomes increasingly routine — and often invisible — the ethical task is no longer simply to decide whether AI should be used, but to determine how its use can be governed in ways that protect epistemic integrity and sustain trust in the medical literature.

Not a bad opening paragraph? It was created using ChatGPT 5.2 with the prompt ‘write an opening paragraph for an editorial on the ethics of AI use in medical research and publishing‘. It is included here deliberately to illustrate a point. We agree with it, we could plausibly have written it, and we strongly suspect it would not have been detected as AI-generated. Empirical studies of AI detection tools suggest they perform only marginally better than chance in identifying lightly edited AI-generated academic prose, with false positive rates high enough to raise concerns about unfair accusations.^3^ So, is there a problem?

The core ethical issue with use of AI, specifically large language models (LLMs) like ChatGPT in medical research and publishing is not primarily about accuracy but about automation of judgement, in-built biases, epistemic integrity (as ChatGPT phrased it in ‘our’ first paragraph), and structural dependence. Medical researchers are using AI for a range of different purposes. Bibliometric analyses indicate rapid growth in publications mentioning ‘large language models‘ or ‘generative AI‘ in biomedical contexts since 2022, with exponential year-on-year increases.^4^ Some of these uses, such as checking for errors in research data, helping to write code, or generating lay-friendly research summaries, are often seen as relatively unproblematic. Randomised evaluations in systematic reviewing, for example, have found that AI-assisted abstract screening can reduce workload by up to 40–50% while maintaining high sensitivity, albeit with non-trivial false exclusion rates that still require human oversight.^5^ But even these comparatively ‘simple’ AI tasks often require a degree of human-like judgement.

Where tasks require human-like judgement, the use of AI raises fundamental questions about epistemic integrity. In other words, can we place trust in the use of AI to generate new knowledge? The same task given twice to the same AI system can produce two different outputs. This variability, of course, is also a feature of human reasoning. However, unlike humans, AI systems are not accountable and arrive at outputs through opaque, ‘black-box‘ processes that are not open to scrutiny. Even developers may not be able to fully trace why a particular output was generated. Moreover, well-documented phenomena such as ‘hallucination‘ persist: studies evaluating LLM-generated medical references have reported fabricated or inaccurate citations in 20–60% of cases, depending on prompt design and domain specificity.^6^ In clinical question-answering tasks, accuracy rates vary widely, often ranging from 60–85%, with performance highly sensitive to phrasing and context.^7^ Such variability is not trivial in domains where small inaccuracies can have outsized consequences.

As many qualitative researchers will tell you, knowledge does not exist in a vacuum. Without going into a detailed ontological or epistemological argument, it is widely accepted that much (arguably all) new knowledge generated relies on pre-existing human concepts or values. How this is done is complex and, despite the goal of science to minimise bias, this can only be achieved to an extent. LLMs do not think or understand; they are systems for synthetic text generation. When they appear to ‘understand’ or ‘generate new knowledge’, they are recombining existing human constructs into highly plausible outputs based on prior data. Like humans using their accumulated experience and knowledge to create, LLMs draw from a huge repository of internet data as the basis for their responses. Importantly, the data on which contemporary AI systems are trained are predominantly Anglophone and statistically skewed toward Americo-European perspectives, reflecting both the structure of the internet and existing published literature. Estimates suggest that over half of widely used training datasets are in English, with comparatively limited representation of low- and middle-income country contexts.^8^ If medical publishing already exhibits geographic and linguistic inequities — with authors from high-income countries accounting for a disproportionate share of indexed publications — then AI systems trained on this corpus risk amplifying those imbalances, subtly reinforcing dominant paradigms while marginalising alternative epistemologies.

These concerns are unfolding alongside a broader, and often under-examined, shift in research culture. The adoption of AI is accelerating, frequently under the radar, driven not only by individual researchers but by institutional pressures. Global investment in AI technologies now runs into hundreds of billions of dollars annually, and higher education institutions increasingly integrate AI strategies into their research plans. Grant calls explicitly referencing AI have increased markedly in the past three years. Universities, funders, and research organisations increasingly promote AI as a marker of efficiency, competitiveness, and innovation. In this environment, the question subtly shifts from “should we use AI here?” to “why aren’t we using AI?”, creating a default expectation of uptake rather than a context-sensitive evaluation of appropriateness. As AI becomes embedded in research and publishing workflows, there is a risk of structural dependence, with processes, expectations, and productivity norms increasingly designed around its use. If peer reviewers come to expect highly polished, linguistically sophisticated prose, authors who choose not to use AI, or who lack access to premium tools, may be disadvantaged. In parallel, there is a risk of a publishing culture in which fluency, coherence, and polish are given more importance over accuracy, reasoning, and whether something is worthy of being believed; where sounding right matters more than being right.

Addressing the ethical challenges posed by AI in medical research and publishing requires both technical safeguards and a deeper cultural shift. From a technical safeguarding standpoint, we need to go beyond generic AI use disclosures to requiring detailed disclosures. Several leading journals and publishers, including BJGP Open, have now issued AI policies,^9^ yet requirements vary widely and enforcement remains inconsistent. Robust AI use policies should require authors to explicitly state the way in which AI is used throughout the research process; statements such as ‘AI was used in the preparation of this manuscript‘ are insufficient. Editors and peer reviewers also have a responsibility to challenge unclear, inadequate, or inconsistent disclosures of AI use, and to raise concerns where AI involvement is suspected but not transparently reported. Each point in the research lifecycle should require mandatory human verification and explicit responsibility taking. Authors should specify which AI systems were used, for what purposes, with what prompts or parameters, and in ways that allow others to understand, scrutinise, and, where relevant, reproduce the process. Without this level of specificity, accountability is diluted and ethical oversight becomes largely symbolic.

While tools, policies, and detailed AI use statements are necessary, they are not sufficient to manage the potential harms of AI in research. Ethical use cannot be reduced to compliance checklists. It requires active deliberation led by senior academics and institutions that creates genuine space for discussion about whether and why AI should be used in particular contexts. Some research activities, especially those involving significant levels of interpretation, value judgements, or vulnerable populations, may warrant restraint or outright rejection of AI assistance. A healthy research culture must make such rejection not only permissible but legitimate, rather than framing it as technophobic or regressive.

This cultural shift must be accompanied by clear responsibility structures and coherent rules governing AI use, alongside alignment between academic journals and research institutions. Fragmented standards risk confusion and loopholes, where researchers navigate towards the least restrictive requirements. Shared expectations across journals and institutions would help reinforce norms of transparency, accountability, and ethical reflection. Professional bodies and international organisations could play a coordinating role in developing consensus guidance, analogous to existing reporting standards in clinical research. Ultimately, responsible AI use in research is not primarily a technical problem but a cultural one, demanding leadership, clarity of responsibility, and the courage to say no when AI use undermines the values of scholarship.
